# Advances in high‐resolution mass spectrometry applied to pharmaceuticals in 2020: A whole new age of information

**DOI:** 10.1002/ansa.202000149

**Published:** 2021-01-29

**Authors:** Caroline Géhin, Stephen W. Holman

**Affiliations:** ^1^ Chemical Development Pharmaceutical Technology & Development Operations, AstraZeneca Macclesfield UK

**Keywords:** high‐resolution, HRMS, mass spectrometry, MS, pharmaceutical, pharma

## Abstract

Continuous improvements in mass spectrometry (MS) have resulted in the widespread availability and adoption of high‐resolution mass spectrometry (HRMS) across laboratories worldwide. The capabilities and the associated advantages of HRMS make it an invaluable analytical tool for analyte characterization, screening, and quantification methodologies for a wide scope of applications across pharmaceutical development. These applications include drug discovery, product characterizations of both small molecules and novel drug modalities, *in vitro* and *in vivo* metabolism studies, post‐approval quality control, and pharmacovigilance. This review gives an overview of the current capabilities of HRMS and its pharmaceutical applications in 2020, and provides a perspective on the future of HRMS within the pharmaceutical industry.

## 1 INTRODUCTION

Mass spectrometry (MS) is an analytical technique that creates gas‐phase ions from atoms or molecules and measures their mass‐to‐charge ratios (*m/z*).^1^ Mass spectrometers discriminate between ions of different *m/z* by subjecting them to constant, pulsed, or periodically time‐varying electric and/or magnetic fields.^2^ Nowadays, mass spectrometers have become a staple in analytical laboratories across the world, becoming one of the most powerful and versatile techniques, capable of (but not limited to): (i) molecular mass, elemental and isotopic composition determinations, (ii) structural elucidation, and (iii) quantification.

The invention of mass spectrometry dates back to 1897 when Sir Joseph John Thomson and his co‐workers discovered the electron and determined its *m/z*, which awarded him the Nobel Prize for Physics in 1906.^1^, ^3^, ^4^ The first mass spectrometer, originally called a “parabola spectrograph”, was constructed in 1912.^5^, ^6^ It was not until the 1940‐1950s that MS started to spread into industry due to the general commercial availability of instruments, the conception of time‐of‐flight (TOF; 1946^1^, ^3^) and quadrupole (Q; 1953^1^, ^3^) instrumentation, and the coupling to gas chromatography (GC; 1956^6^, ^7^).^1^, ^3^ The coupling to liquid chromatography (LC) was first performed in the 1970s and would further revolutionize MS use.^6^, ^8^ With the continued development and technological advances of MS spanning more than a century, many instruments are currently available that offer different performance characteristics, such as spectral acquisition rates, sensitivities, mass accuracies, resolving powers, and thereby resolutions. The definitions for mass accuracy, resolving power, and resolution are given below as they are key to understanding mass spectrometers and the data generated from them.

$$\mathrm{Resolutio}n^{9}:\;R\;=\frac{m/z}{\Delta \left(m/z\right)}\;$$

*The observed m/z value divided by the smallest difference Δ(m/z) for two ions that can be separated. The m/z is of the ion of interest and the Δ(m/z) is either the peak width at half maximum (peak width definition; FWHM) or the spacing between two equal intensity peaks with a valley between them no more than 10% of their height (10% valley definition). The latter definition is almost exclusively reserved for sector mass spectrometers and seldomly used*.

Resolving power^9^:

*The measure of the ability of a mass spectrometer to provide a specified value of resolution*.

Using an instrument with a high resolving power improves the ability to record a given ion without interference. Therefore, when centring the data,^10^ the resultant signal is more likely to accurately represent just that one analyte, and with good calibration, it will give an accurate *m/z*, with mass accuracy defined as:

Mass accuracy^1^:

*The error limit within which a mass spectrometer can measure an ion's m/z value relative to its theoretical (exact mass) value*.

The terms resolving power and resolution are often used interchangeably despite portraying different pieces of information, where resolving power defines an instrument's capability and resolution defines the output data quality.

Mass spectrometers can be broadly classified as either low‐resolution or high‐resolution based on their resolving powers, where one commonly used definition is that high‐resolution mass spectrometers are capable of resolving powers ≥ 10 000 FWHM. For the purposes of this review, papers using mass spectrometers operating at resolving powers ≥ 10 000 FWHM will be considered.

Low‐resolution mass spectrometers (LRMS), such as quadrupoles and ion traps, generally measure the *m/z* value to the nearest integer, or nominal mass; whereas, high‐resolution mass spectrometers (HRMS), such as the TOF and Fourier transform (FT)‐based mass spectrometers, provide information on the fractional mass with up to 4‐5 decimals. Table 1 shows a comparison of typical performance characteristics of some commonly used mass spectrometers.^1^, ^11^, ^12^, ^13^

**TABLE 1:** Comparison of typical performance characteristics of commonly used mass spectrometers. Table generated using data from^1^, ^11^, ^12^, ^13^

Mass analyzer type^a^	Resolving power (FWHM) [x10^3^]	Mass accuracy (ppm)	*m/z* range (upper limit) [x10^3^]	Price
Quadrupole (Q)	< 5	> 100	2‐4	Lower
Ion trap (IT)	< 5	< 30	4‐20	Lower
TOF^b^	10‐60	0.5‐5	100	Moderate
Orbitrap	120‐1000	0.5‐5	20	Higher
FT‐ICR	100‐10 000	0.05‐1	30	High

^a^ TOF, Orbitrap and ICR include common hybrid configurations with Q or LIT as the first mass analyzer.

^b^ Values quoted for reflectron‐TOF instruments. Linear TOFs capable of greater *m*/*z* ranges (up to 500 000 in practice, and theoretically limitless) with lower resolving powers.

**FIGURE 1:**
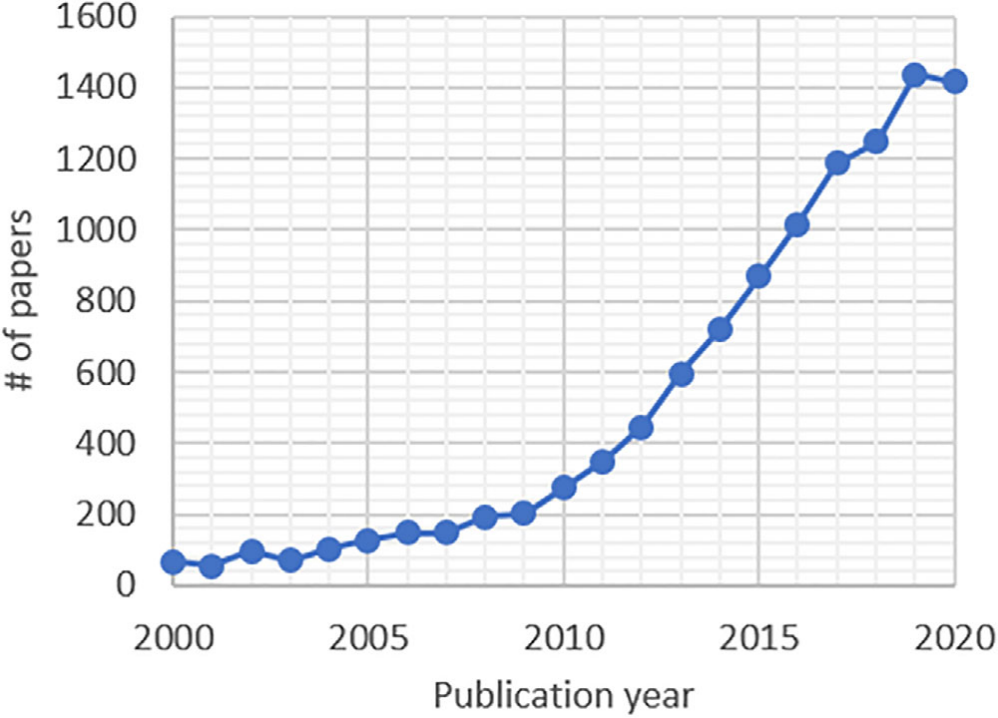
Number of peer‐reviewed papers published annually between 2000 and 2020 using HRMS (Data generated using Scopus, search term “high resolution mass spec^*^”)

HRMS instrumentation is relatively expensive, but it comes with considerable advantages for analytical methodologies in comparison to LRMS, such as (i) the possibility of unequivocal determination of elemental compositions via accurate mass measurements, (ii) improvement in signal‐to‐background ratios as interferences can be removed from the target analyte's signal, and (iii) wide mass ranges that allow the analysis of large, complex molecules. With these advantages, it is of no surprise that the applications of HRMS have rapidly grown since 2000, reflected by the number of papers published (Figure 1).

For pharmaceutical analyses, HRMS combined with a chromatographic separation is an incredibly powerful tool for the monitoring, identification, and characterisation of many molecules including impurities, degradation products, and metabolites. This supports a variety of pharmaceutical applications, extending across drug discovery, development, and quality control.^14^, ^15^ This review discusses selected work published in 2020 using HRMS for pharmaceutical applications to identify current key trends and offer a perspective on the future of HRMS.

## 2 DRUG DISCOVERY AND DEVELOPMENT

The process of discovering and developing a new medicine for market approval typically takes over 12 years with an estimated cost of $2.6 billion.^16^, ^17^ This is a resource‐intensive and time‐consuming process with an incredibly high attrition rate, such that the overall failure rate in drug development is over 96%.^18^ The high development costs often lead to inflated prices of the few successful medicines,^18^ which makes them less accessible to patients and healthcare systems. Development failures can lead to site closures and job losses. This shows the critical need to drive efficiency through tackling the challenges facing pharmaceutical drug development.

The main reason for drug development failure is lack of efficacy, followed by poor pharmacokinetics and toxicity.^18^ These can be attributed to a drug development system flaw, whereby preclinical studies using *in vitro* systems and *in vivo* animal models are unable to accurately predict drug behaviour in human studies.^17^, ^18^ These simpler experimental designs neglect the complexities of biological systems. The difficulties of this transition and getting them approved is well known by the Food and Drug Administration (FDA) and appropriately called the “Valley of Death".^16^, ^19^ Potential improvements to this area include the implementation of less complex biological systems rather than isolated cell lines or cells for the drug screening, better phenotyping of patients, and the application of genomics.^16^, ^18^ They offer invaluable information with regards to a drug's pharmacokinetics, pharmacodynamics, and metabolism.

The advances in MS technology directly impact drug discovery and development as the technique is already qualitatively and quantitatively used to study the parent drug, drug metabolites, and biomarkers in various systems.^14^, ^20^ The advantages of HRMS *vida supra* make it more amenable to where drug development is headed, *i.e*. the shift from isolated analysis to whole systems analysis. This approach and the use of HRMS should produce more complex, yet meaningful data that should enable the deeper and more comprehensive understanding of the biological systems impacted by a drug; therefore, a more complete picture of a drug's efficacy and toxicity. With this information, the detection of drug failures should occur earlier in the drug development process, potentially lowering the overall drug development costs, and further decreasing the post‐approval prices of future medicines and the attrition rate.

## 3 NEW DRUG MODALITIES AND HRMS CHARACTERIZATION

With our ever‐growing understanding of biological and cellular systems, a range of new biological drug targets, such as protein‐protein and protein‐nucleic acid interactions, are of interest to tackle many diseases that were previously thought to be intractable.^21^ Generally, small molecules cannot target these and therefore, the pharmaceutical industry is looking beyond small molecules for new “modalities” (a term being increasingly used within the pharmaceutical industry).^21^, ^22^ These include biologics and synthetic molecules of different shapes and sizes, such as peptides, proteins, oligonucleotides, hybrids, molecular conjugates, and macrocycles. These modalities have been steadily progressed and developed over several decades, where some have reached clinical development and obtained regulatory approvals^22^; however, they present novel analytical challenges that both academia and industry are rapidly tackling. HRMS greatly complements the analytical toolbox to study these new modalities, especially in terms of their characterisation and structural elucidation.

At Janssen pharmaceutical laboratories, an automated open‐access ultra‐high performance liquid chromatography (UHPLC)‐Q‐TOF‐MS instrument has been in use for the past three years for a large diversity of compound classes.^23^ There is the potential that HRMS instrumentation will gradually replace the LRMS open‐access systems currently used, providing more information‐rich MS spectra in the same short timeframe. Fontana *et al*. developed several methods using different electrospray ionization (ESI) conditions and different collision energies to enable the analysis of most medicinal chemistry compounds. Their “universal MS ESI+ method” produces high quality spectra with good S/N and mass accuracies within 1 mDa, enabling the automatic identifications and reporting of 98% of the ionised compounds. This methodology is being used by chemist end‐users, working unattended, enabling them to perform HRMS characterisations of synthetic compounds in 95% of the cases with no assistance from the analytical team.^23^

**FIGURE 2:**
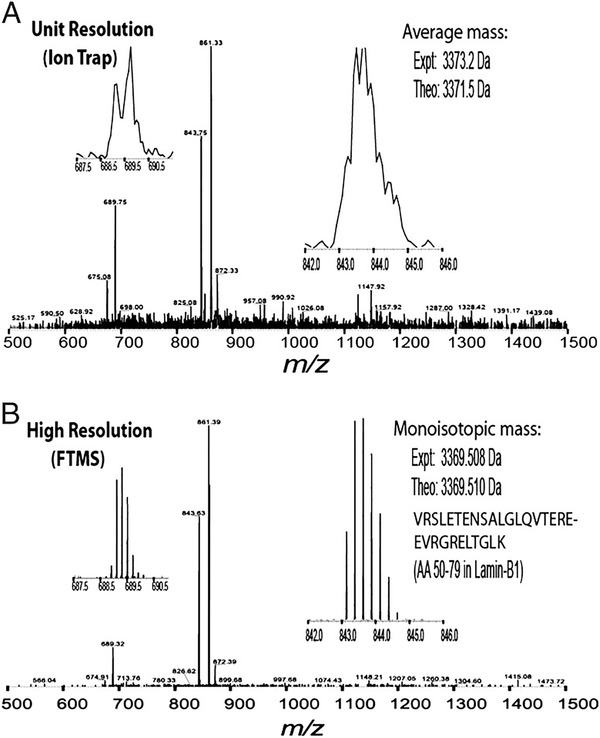
Experimental data of a mixture of peptides analysed at (A) low‐resolution and (B) high‐resolution. Expanded spectra are shown on the sides for individual peptides, where the right inset shows an unmodified Lys‐C peptide that is 30 residues long. The differences seen between these resolutions are clear where at high‐resolution, the natural ^13^C isotopes are resolved due to the high resolving power, accurate mass measurements to three decimal places are generated that will aid analyte identification and characterisation, and the mass spectrum is visually ‘cleaner’ due to the removal of interferences. On the other hand, the low‐resolution data shows a broad 2 Da peak with an inaccurate experimental average mass, limited isotope information, and a noisy baseline, which will provide low confidence analyte identification. Reprinted with a permission from^24^ Copyright (2008) National Academy of Sciences, U.S.A.

Generally, MS characterizes molecules in two ways: (i) as intact masses and (ii) as dissociated structure‐specific fragments. Prior to HRMS, LRMS data had to be relied on for species identification, where using the unit mass values often resulted in ambiguous assignments and relied heavily on fragment interpretation. HRMS, on the other hand, has the ability to enable high confidence species’ identification through accurate mass measurements and relative isotopic abundances, detect larger *m/z* values due to its wider mass range, and the ability to resolve isotopes and related impurities better. An example of the differences in information between LRMS and HRMS analyses is shown in Figure 2. This figure depicts how the high resolving power significantly separated the components and removed interferences, and further the accurate mass measurement (shown here to three decimal places) will allow confident identifications for most of the ions of interest.^24^ Notably, the low‐resolution spectrum was unable to achieve isotopic resolution and therefore has changed reporting from monoisotopic to average mass.

For the analysis of large molecules (*i.e*. ≥1 kDa), which is becoming more frequent for the new modalities, HRMS offers significant advantages, particularly concerning the measurement of isotope patterns. Firstly, using ESI, molecules can adopt multiple charges. Using the resolution equation above, it can be seen that as the charge (*z*) increases and *Δ(m/z)* remains the same, the gap between neighboring isotopic peaks halves and therefore would need twice the resolving power to maintain the same resolution.^1^ In this case, LRMS might not have sufficient resolving power to show this isotopic data.

**FIGURE 3:**
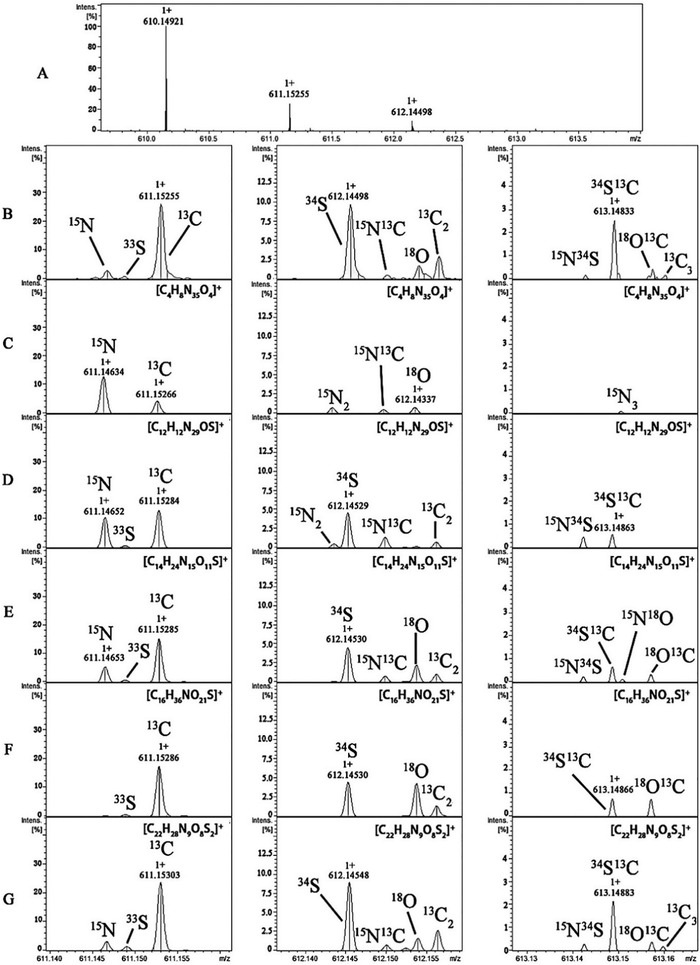
Elemental composition determination through the comparison of experimental and theoretical IFSs: (A) the experimental IFS of the *m/z* 610.14921 ion, (B) the expanded IFS spectrum of (A), and five theoretical IFSs of (C) [C_4_H_8_N_35_O_4_]^+^, (D) [C_12_H_12_N_29_OS]^+^, (E) [C_14_H_24_N_15_O_11_S]^+^, (F) [C_16_H_36_NO_21_S]^+^, and (G) [C_22_H_28_N_9_O_8_S_2_]^+^. From this visual comparison that is only possible using ultra‐high‐resolution MS, the formula of the *m/z* 610.14921 ion was assigned to (G) [C_22_H_28_N_9_O_8_S_2_]^+^. Reprinted with a permission from^61^

Secondly, with increasing molecular mass, the number of possible elemental formulae increases for a given accurate mass measurement.^1^ Mass spectrometers capable of high mass accuracies will significantly decrease the number of overall possible elemental compositions compared to low mass accuracy data; however, for large molecules with higher *m*/*z* values, many formulae will still be possible regardless of mass accuracy such that an analyst will not be able to assign the elemental composition using this alone.^25^, ^26^ In this case, the use of isotopic fine structure (IFS) is an invaluable, complementary approach for data interpretation that instead relies on the mass spectrometer's resolving power. A mass spectrometer with a resolving power ≥ 300 000 FWHM (termed "ultra‐high‐resolution mass spectrometry"; typically FT‐MS instrumentation) is required for routine IFSs.^25^ IFS is the mass spectrometric fingerprint arising from the naturally occurring isotopes within a molecule being measured to high‐resolution.^26^, ^27^ An example of this is shown in Figure 3, where a single unresolved peak 1 *m/z* unit greater than the monoisotopic peak has been resolved into the separate ^13^C, ^15^N, and ^33^S isotopologues. In principle, the natural abundances of elemental isotopes are well understood and therefore through analyzing the IFSs, the number of each element can be readily calculated and therefore be used to deduce the compound's elemental composition.^25^, ^26^, ^27^ This is an emerging area that has seen limited application due to the high costs of ultra‐high‐resolution mass spectrometers, but provides a powerful new approach to elemental composition determination when sufficient mass resolution is achievable.

## 4 HRMS SCREENING

The advances in HRMS has enabled analysts to capture data that was previously unattainable, where (i) the high accurate mass measurements allow high confidence analyte identification, (ii) the high ion selectivity increases sensitivity and detectability of analytes in complex matrices, and (iii) the wide mass ranges allow a greater molecular weight range of analytes to be detected. This means that using HRMS, real unbiased wide‐scope multi‐residue screening methods, capable of simultaneously analyzing thousands of compounds at once, can be developed, which makes the technique ideal for both targeted and non‐targeted screening methods. This allows scientists to readily capture data or 'snapshots' of complex samples that was not possible previously using LRMS.

**FIGURE 4:**
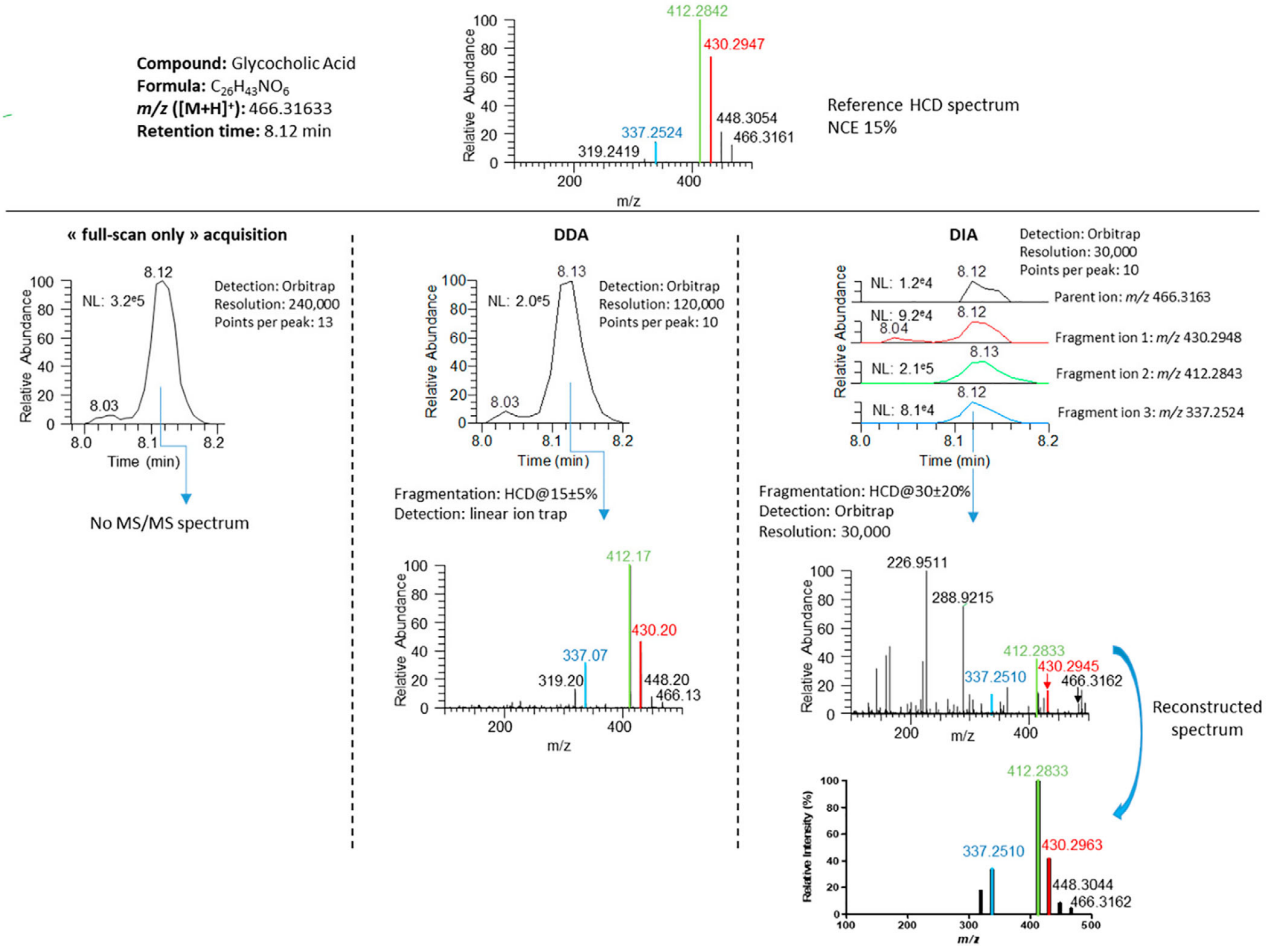
Data of glycocholic acid generated using three different MS and MS/MS acquisition modes: full scan, DDA, and DIA. Full scan mode can be seen to provide limited analyte information due to the lack of fragmentation MS/MS data in MS^2^. Comparison of DDA and DIA spectra shows the clear difference in the number of analysed ions in both MS^1^ and MS^2^, where the DDA collected and captured the MS/MS data for the defined precursor ion only and the DIA has collected data for all the ions in the *m/z* window, which has resulted in ‘busier’ but more comprehensive mass spectra. Reprinted with a permission from^28^

The acquisition modes generally used for screening methods to generate tandem mass spectrometry (MS/MS) data are called data‐dependent or data‐independent acquisition (DDA and DIA, respectively). In the DDA workflow, precursor ions are either automatically selected typically based on intensity by the instrument or predefined by the analyst in MS^1^, followed by the fragments of the ions being analyzed in MS^2^. In contrast, in the DIA workflow, either wide, consecutive *m/z* isolation windows (typically between 10‐50 *m/z*) are created to cover the entire *m/z* range or the whole *m/z* range is analyzed at once in MS^1^, systematically collecting MS/MS data for every ion detected in MS^2^.^28^, ^29^ The differences in resulting MS/MS spectra for these acquisition modes can be seen in Figure 4. The DDA and DIA modes generate different data sets with their own specific strengths, but also drawbacks, that are important to know before analysis. DDA incorporates significant data bias as the precursor ion selection intentionally dismisses other ions, therefore generating mass spectra that are not representative of the whole sample composition. This makes DDA less sensitive overall (as precursor ion selection is typically intensity‐based), but the resulting MS/MS spectra tend to be 'cleaner' and easier to interpret.^28^, ^29^ Further, precursor ion selection is stochastic, so repeatability of data is impacted. On the other hand, DIA does not introduce bias as it detects and fragments all ions in a sample. DIA offers a more comprehensive and repeatable analysis of a sample by collecting data for a wide range of known and unknown ions, which generally generates more complex spectra that can be harder to interpret, but will be more suited for semi‐targeted and non‐targeted screenings.

Non‐targeted HRMS screenings present multiple challenges, such as complex data mining and analyte quantification without authentic standards. Firstly, with large data sets comes an equally large number of ways to process the data. Almost every paper involved in this review has used different *post hoc* data manipulation tools, such as background subtraction, mass defect filtering, and batch effect correction, and software, such as MZmine, Metworks, MetabolitePilot, MetaboAnalystR, and Compound Discoverer. The way data is processed will introduce bias and therefore in activities such as environmental monitoring and doping analysis, *i.e*. those that are global collaborative efforts, there is an important need for definition and harmonization in data processing and workflows. An exciting area to watch in the coming years is the development of HRMS workflows, the ‘coupling’ of HRMS non‐targeted screening data processing with literature mining, and the increased use of artificial intelligence and machine learning for data interpretation.

Secondly, the difficulty of quantification without standards is that different compounds ionize with different efficiencies in the ionization source and are impacted by changes in measurement and instrument conditions. Liigand *et al*. proposed a semi‐quantification approach for non‐targeted analyses through using predicted ionization efficiency values for different compound classes. The work investigated both positive and negative ESI conditions, more than 100 eluent compositions and covered over 450 compounds, and the results suggest that this approach can be transferred between different eluents, LC setups, and instruments, and can be used for real samples. This has been validated for over 35 compounds, including pesticides and mycotoxins, in cereal samples.^30^ This quantification approach could lead to higher work throughput, cost savings, and potentially wider usage as the limitation of previously needing standards, which could have been a financial barrier, is removed. It will be interesting to see how this area develops in the coming years and whether it is applicable for the quantification of pharmaceuticals.

## 5 HRMS APPLICATIONS IN 2020

### 5.1 Proteins and peptides

Protein characterization was once dominated by classical techniques, such as optical spectroscopy, light scattering, ultracentrifugation, calorimetry, SDS‐PAGE, and size exclusion chromatography, due to the limitations imposed by the analytes’ large molecular weights that offered little structural information.^31^, ^32^ The application of MS to protein characterization heralded the large‐scale analysis of many proteins in a single analysis, termed “proteomics”. Initially performed by LRMS using ion trap mass spectrometers, the growth in HRMS has enabled new insights for characterization, peptide sequence determination, post‐translational modifications, and higher order structure (particularly through the use of native MS).^31^, ^32^

Native MS is a particular ESI approach where the biological analytes are sprayed from a non‐denaturing solvent in order to conserve noncovalent interactions in the gas phase for analysis, *i.e*. protein assemblies remain in their tertiary and quaternary states.^33^ Native MS is typically performed using nano‐ESI due to its low sample consumption, more uniform response factors, and higher tolerances to salts and buffers.^33^ These analyses offer insight on subunit stoichiometry, binding partners, protein complex topology, protein dynamics, and binding affinities. Native MS was coined back in 2004^33^ and its applications have grown since for the analyses of protein assemblies, DNA‐protein and RNA‐protein assemblies with the aim to define structure‐function relationships. This information is important for our understanding of protein interactions, structural biology, drug design, and disease diagnostics.^31^

Bekker‐Jensen and co‐workers aimed to tackle the time‐consuming nature of deep proteome profiling of human cells, which typically takes days of MS measurement time, by developing a rapid method for high‐throughput, large‐scale analyses. For this, the group used a high‐field asymmetric waveform ion mobility (FAIMS) UHPLC‐Q‐Orbitrap‐MS DIA method, such that the quantification of > 5000 proteins with short LC gradients for up to 60 samples per day was achievable. FAIMS is an atmospheric pressure ion mobility technique that can separate or select gas‐phase ions based on a combination of factors, such as charge state, shape, conformation, and size, using strong and weak electric fields.^34^ The application of FAIMS in the work of Bekker‐Jensen and co‐workers removed the majority of singly‐charged peptides and background ions, thereby favoring transmission of multiply‐charged peptide species into the mass analyser. This simplified the resulting mass spectra, prevented filling the Orbitrap's C‐trap with ions for which no useful information would be obtained, and increased the efficiency of MS/MS through the analysis of multiply‐charged peptides. Through the use of FAIMS, the group acquired higher sensitivity and greater proteome coverage, allowing the identification of >1000 proteins using a 5 ng sample of HeLa digest. To demonstrate instrument versatility, the group recorded an organ‐wide map of proteome expression across 12 rat tissues quantified by tandem mass tags and label‐free quantification using DIA‐FAIMS to depths of >10 000 proteins. The group further compiled a general rodent spectral library consisting of 213 000 unique peptides covering 12 909 protein‐coding genes for the larger proteomics community.^35^ This methodology has the potential to expand the use of chemoproteomics^36^ (the investigation of the effect of small molecule on the proteome of biological systems) as the higher‐throughput of large‐scale proteome measurements is more compatible with the time‐scales of pharmaceutical drug discovery.

Al Matari *et al*. developed the first nano‐LC‐Q‐Orbitrap‐MS method for the identification and semi‐relative quantification of intact α‐subunit glycoforms of the targeted proteins of human chorionic gonadotropin (hCG) and follicle‐stimulating hormone (FSH). This method allowed the detection of both major and minor isoforms (>30 hCGα and 30 FSHα glycoforms) with high mass accuracy, minimal sample preparation, and good repeatability.^37^ This work highlights the potential of nano‐LC‐HRMS for fast glycoprofiling methods, which would prove useful for the pharmaceutical development of glycoprotein‐based drugs.

Cyclic peptide MS characterization is not as well understood as linear peptides and therefore, as a result, there are fewer *in silico* tools available. MetabolitePilot Software 2.0 uses automated LC‐HRMS processing of both targeted and non‐targeted screening data sets to detect and structurally characterize therapeutic peptides, including non‐linear, cross‐linked, and cyclic peptides, and any associated metabolites. Yao *et al*. evaluated this software by studying insulin and atrial natriuretic peptide (ANP) using UHPLC‐Q‐TOF‐MS instrumentation. The results from the study demonstrate the accurate prediction and rapid identifications of cyclic peptide metabolite profiling by the software for both post‐hydrolysis and *in vitro* samples.^38^ Accurate software is key to maximize work throughput to get medicines to market faster, and therefore this evaluation will prove useful across drug development.

### 5.2 Small molecules

Protein degraders, commonly known as proteolysis targeting chimeras (PROTACs), are small molecule inhibitors that block the activities of intracellular target proteins and interfere with signaling pathways.^39^, ^40^ This is an emerging modality that has seen limited growth due to the complexities of the PROTAC‐mediated protein interactions that involve binary and ternary interactions between the components.^39^, ^40^ Beveridge *et al*. used native nano‐ESI‐Q‐TOF‐MS to effectively study PROTAC‐mediated protein complexes in a label‐free manner. The use of native MS was demonstrated to be capable of the characterization of PROTAC‐protein complexes, semi‐quantification of their equilibria, insight into PROTAC specificities for substrate proteins and PROTAC cooperativity, and competitive experiments.^40^ This work highlights the potential of native‐HRMS for high‐throughput methods in PROTACs development.

Monomethyl auristatin E (MMAE) is an antimitotic agent used as a payload of an antibody‐drug conjugate, which shows potent activity in clinical studies against various lymphomas, leukemia, and solid tumors. Lee *et al*. developed and validated a UHPLC‐Q‐TOF‐MS method for the quantification of MMAE and its preclinical pharmacokinetics to study its short‐term and long‐term stability profiles and to evaluate the results from a pharmacokinetic study in rats as a preclinical animal model. The HRMS approach for this study helped generate a comprehensive understanding of the pharmacokinetic characteristic of this specific payload and could be applied generally to the development of other antibody‐drug conjugates.^41^

Baclofen is a GABA_B_ receptor agonist and the only small molecule drug currently approved for the treatment of spasticity in multiple sclerosis. A therapeutic alternative to direct GABA_B_ agonism would be positive allosteric modulation of the GABA_B_ receptor of which a molecule COR659 was identified to be a potential candidate. Ferlenghi's group studied COR659's phase I *in vitro* and *in vivo* metabolism in liver microsomes and rat models, respectively, and pharmacokinetics aided by UHPLC‐Q‐Orbitrap‐MS instrumentation. Following this, novel COR659 analogues were synthesized to improve metabolic stability and *in vivo* potency. This allowed an in‐depth study of the differences seen between *in vivo* and *in vitro* samples as well as attribute pharmacokinetic behaviors to specific chemical functionalities, identify molecular soft spots, and their predicted interactions.^42^

Similarly, Yu *et al*. developed and validated a high‐throughput and selective UHPLC‐IT‐TOF‐MS method for the quantification of T0901317, a synthetic liver X receptor (LXR) agonist, in mouse serum, liver, and brain samples. This is the first time the pharmacokinetics of this drug has been reported across biological samples. The findings will help develop novel, efficacious experimental strategies for tissue‐specific LXRs function studies by providing accurate dosing and sampling protocols,^43^ but also demonstrates the strength of HRMS for analyzing complex sample matrices.

### 5.3 COVID‐19

Severe acute respiratory syndrome coronavirus 2 (SARS‐CoV‐2) is a positive‐sense RNA virus that triggered the worldwide COVID‐19 pandemic in 2019‐2020 with more than 35 million people infected and more than 1 million deaths as recorded by the World Health Organization (as of Oct 12, 2020).^44^ It is characterized by acute respiratory distress and excessive inflammation capable of inducing respiratory failure, multiple organ failure, and death.^45^, ^46^ Due to the asymptomatic nature of SARS‐CoV‐2 infection and its high incident rate, public health strategies are struggling to contain it; therefore, there is an urgent need to develop vaccines and drug therapies to protect against and treat COVID‐19. For this purpose, several groups have used HRMS to identify potential drug candidates for COVID‐19.

Bouhaddou *et al*. performed a non‐targeted quantitative phosphoproteomics screen using UHPLC‐Q‐Orbitrap‐MS on Vero E6 cells to study how cellular pathways and processes are hijacked by SARS‐CoV‐2 infection over time by monitoring the changes in phosphorylation and protein abundance. This allowed the study of virus‐host protein‐protein interactions, where altered kinase activity was observed. Kinases are ideal drug targets and with this in‐depth understanding of SARS‐CoV‐2 infection, the group identified 87 compounds, some of which are FDA‐approved or in clinical trials for other indications, to potentially treat SARS‐CoV‐2 infection.^47^ Similarly, Gordon *et al*. used UHPLC‐Q‐Orbitrap‐MS to identify with high confidence 332 SARS‐CoV‐2 virus‐host protein‐protein interactions that are linked to multiple biological processes, such as protein trafficking, translation, transcription, and ubiquitination regulation. With the understanding of the elaborate virus‐host interaction networks, the team identified 69 ligands, which again included FDA‐approved drugs, clinical, and preclinical compounds that already target these interactions, and therefore have a potential to combat SARS‐CoV‐2 infection.^48^ Both works present an alternative, innovative approach to drug discovery on a cellular level, which has enabled the speedy identifications through literature mining of potential therapeutic drugs that allows the rapid prioritization and repurposing of drugs.

Qingfei Paidu Decoction (QFPD) is a Chinese medicine clinically used to treat COVID‐19 patients in China; however, the chemical composition and its pharmacological mechanism are not understood. Yang *et al*. used a UHPLC‐Q‐TOF‐MS to identify a total of 129 compounds in QFPD. Through *in silico* molecular and therapeutic target networking of the MS data, it was found that Ma Xing Shi Gan Decoction (MXDGD), a core component of QFPD, may target the toll‐like signaling pathway. Transcriptomic analysis of MXDGD effects in a rat model of pneumonia suggested that MXSGD regulated the coagulation system in the inflammatory state, which benefits QFPD to intervene with the inflammatory or cytokine storm caused by COVID‐19.^49^

### 5.4 Herbal medicines

Herbal medicines and extracts, including traditional Chinese medicines, have been in medical use for thousands of years.^50^, ^51^ These medicines are composed of a complex mixture of organic, plant materials that could stem from the leaves, stems, flowers, roots, or seeds, for therapeutic activity through the synergistic effects of numerous active components with individual mechanisms for many drug targets. Understandably, the quality and safety controls for these medicines significantly diverge from those of typical pharmaceutical drug development, and therefore, with the steadily growing interest in herbal medicines due the global popularization of natural and holistic treatments,^48^, ^49^ there are key concerns regarding efficacy, safety and legality.^51^ These concerns limit the international applications and distribution of herbal medicines worldwide.^50^ In recent years, LC‐HRMS has been increasingly used for the rapid chemical profiling of these complicated herbal medicines due to its strength in characterisation and screening methodologies. These analytical methodologies could be important for regulatory quality assessments of these medicines.

Walnut leaf is a hypoglycemic Chinese herbal medicine used for the treatment of diabetes mellitus (DM).^52^ The active components and mechanisms are not clear, and therefore Liu *et al*. studied them using UHPLC‐Q‐Orbitrap‐MS and identified 130 components through accurate mass measurements, MS/MS data, and literature mining. Through network pharmacology analysis, 38 of these components were considered bioactive with 8 key active components sharing high similarities and common targets with FDA‐approved drugs for DM. The compound‐target interactions were further validated through molecular docking analysis.^52^

In a similar fashion, Gao and co‐workers studied Dingkun Dan, a Chinese herbal medicine for the treatment of many gynecological diseases.^53^ Using UHPLC‐Q‐Orbitrap‐MS in DDA mode, 121 components were characterized by accurate mass measurements, MS/MS data, and literature mining. Through network pharmacology, 76 compounds were considered bioactive and further investigated using molecular docking analysis.^53^

*Arnebiae Radix* is a Chinese herbal ointment that has been used for several hundred years for the treatment of infection, inflammation, obesity, and bleeding disease.^54^ Feng *et al*. used UHPLC‐Q‐Orbitrap‐MS together with an integrated data filtering and identification strategy to efficiently identify the chemical constituents of *Arnebiae Radix*. A total of 96 compounds were characterized from 20 sources. Nine of these compounds were detected for the first time.^54^

As an alternative form of drug discovery, several groups analyzed known medicinal herbal extracts to identify lead compounds that then can be isolated for drug development. Peeters *et al*. studied *Herniaria hirusta L*., which is an herbal extract known to treat urinary stones and act as a diuretic, to identify the active compounds responsible for the pharmaceutical effects and the mechanism of action. In this study, a sample of well‐characterized *H. hirusta* extract was investigated in an *in vitro* gastrointestinal model followed by hepatic biotransformation to screen and tentatively identify metabolites formed after *in vitro* biotransformation. The team was able to work backwards from identifying the phase I and II metabolites to determine potential lead compounds to treat urinary stones.^55^

Montone's group developed a data processing workflow specifically dedicated to the untargeted identifications of phytocannabinoids using Compound Discoverer software, where the group has made a database of 533 phytocannabinoid derivatives for ease of identification and greater work efficiency. Of interest, isomers were able to be distinguished through MS/MS interpretation for these compounds by using negative ESI rather than positive ESI. This workflow was trialed on extracts of medicinal cannabis such that 121 phytocannabinoids were readily identified in a single analysis.^56^ This workflow can be used to identify novel bioactive compounds of *Cannabis sativa* and perform comparative studies of medicinal cannabis in circulation.

## 6 POST‐APPROVAL ANALYSES & HRMS QUANTIFICATION

Research on a new medicine does not terminate when it gets market approval. Pharmaceutical companies are required by the FDA to monitor approved medicines for as long as they are on the market and submit periodic reports on safety, efficacy, and tolerability. Companies may also perform additional research to assess the drug against other indications, improve formulations and dosage, and possibly optimize manufacturing routes. HRMS has been applied to many post‐approval drugs in 2020, either to address gaps in drug understanding or to create simpler and/or more comprehensive methods for quality control and monitoring purposes.

For many decades, triple quadrupole systems (QqQ) have been the gold standard for the quantification of drugs and metabolites due to the sensitivity and selectivity offered by selected reaction monitoring (SRM) mode.^57^, ^58^ Due to the advances in HRMS technology, the new generations of TOF and Orbitrap instruments have the performance characteristics required for reliable and routine quantification, *i.e*. sensitivity, dynamic range, accuracy, precision, and reproducibility.^57^, ^58^, ^59^ On top of this, HRMS quantification using targeted approaches has additional advantages over QqQ quantification. One advantage stems from the greater selectivity of HRMS, where with a better separation of ions from interferences, there is a decreased chance of detecting false positives or generating artefactually greater measurements. Another advantage, which has been discussed previously, is the ability to perform the quantification in full‐scan mode, which means data can be captured for all ions in a sample, giving a global picture that is more representative of the sample.^57^, ^58^, ^59^, ^60^ This large, global data set allows retrospective analysis and different kinds of *post hoc* targeted or untargeted data treatments.^59^ To be selective and sensitive, QqQ mass spectrometry is limited to measuring SRM transitions for quantification that require *a priori* knowledge of the precursor and product *m/z* ions to be recorded and MS/MS conditions necessary.^57^, ^58^, ^59^, ^60^

### 6.1 Further drug understanding

Yue *et al*. identified the gap that for the active pharmaceutical ingredient (API) Cefteram pivoxil, there was no impurity profiling report available. Impurity profiling is an important quality control activity as an understanding of a drug's impurities can significantly affect its efficacy and safety, even in small amounts. The group used HPLC‐FT‐ICR‐MS to identify and characterize the impurities of Cefteram pivoxil by analyzing forced degradation samples. Twenty impurities were found through peak picking, where 6 were process‐related impurities and 14 degradation products, and subsequently identified through using accurate mass measurements for elemental compositions, MS/MS fragmentation interpretation, and IFSs (as shown in Figure 3). Thirteen of these impurities were discovered and reported for the first time in this study. IFSs were required to discriminate between elemental compositions as the accurate mass measurements allowed for many possible formulae for the analytes.^61^

Troxipide is a therapeutic agent for gastritis and gastric ulcer.^62^ Detailed studies concerning metabolites, pharmacokinetics, and mechanism are missing thereby limiting the clinical use of the drug. Guo *et al*. identified the 45 phase I and II metabolites of troxipide by using UHPLC‐Q‐TOF‐MS through accurate mass measurements and MS/MS fragmentation interpretation by analyzing plasma, fecal, and urine samples to understand the metabolic pathways of the drug. This is the first time these 45 metabolites have been reported for troxipide thereby filling an information gap.^62^

Melanotan II is a cyclic heptapeptide drug that has been shown to increase insulin sensitivity, increase skin pigmentation, and treat sexual dysfunction in various preclinical and clinical studies.^63^ Of particular interest, Chen *et al*. used both matrix‐assisted laser desorption/ionization (MALDI)‐FT‐ICR‐mass spectrometry imaging (MSI) to study the spatial distribution of the drug and its metabolites in resected organs and whole‐body tissue, and droplet‐based liquid micro‐junction surface sampling‐HPLC‐Q‐Orbitrap‐MS to provide a chromatographic separation, molecular profiling, structural elucidation, and quantification of tissue analytes at the expense of spatial resolution. This work highlights the capabilities of using these label‐free MS techniques, either together or independently, to qualitatively study the pharmacokinetics and pharmacodynamics of a drug to evaluate *in vivo* stability and biodistribution.^63^ However, the lack of chromatographic separation and the influence of matrix effects makes it unlikely that MSI will become a widely used quantitative technique for small molecules in biological matrices.

Demelenne *et al*. studied chemically modified and unmodified oligonucleotides using UHPLC‐drift tube ion‐mobility (DTIMS)‐Q‐TOF‐MS and capillary zone electrophoresis (CZE)‐DTIMS‐Q‐TOF‐MS, where through successful method development, both separation techniques showed strengths in their selectivity for both shortmers and longmers, while the DTIMS provided information about ion conformations and the Q‐TOF‐MS generated accurate mass measurements. DTIMS could determine with good accuracy the number of nucleotides in a given oligonucleotide, while the HRMS data provided the identity of the nucleotides.^64^ This work could prove useful for medicinal oligonucleotide development and quality control.

Radrezza *et al*. studied the intra‐ and extra‐cellular effects induced by low‐molecular‐weight hyaluronic acid (LWM‐HA), an unbranched glycosaminoglycan, in normal human dermal fibroblasts after 24 h treatment at 3 different concentrations of LWM‐HA of 20‐50 kDa. This was achieved through a DDA screening using nano LC‐Q‐Orbitrap‐MS together with network protein analysis to identify and quantify the proteome's changes. Overall, 2328 proteins were identified of which 39 altered significantly in 0.125% LWM‐HA, 149 in 0.25% LWM‐HA, and 496 in 0.50% LWM‐HA.^65^ This work has filled a gap in drug understanding and can be useful for the improvement of existing formulations and personalized treatments.

### 6.2 Quality control and surveillance

Joye's group developed and validated a targeted screening method utilizing UHPLC‐Q‐Orbitrap‐MS for the sensitive and selective quantification of the main 37 drugs present in the context of driving under the influence of drugs using 100 μL of whole blood, as a means to replace the current QqQ methods in use.^59^ This new method is quicker, more efficient, and additionally benefits from low sample consumption. From the same group, another application of UHPLC‐Q‐Orbitrap‐MS has been reported for the quantification of cannabinoids in whole blood.^66^ Similarly, Zhang *et al*. developed and validated a targeted screening method using UHPLC‐Q‐Orbitrap‐MS to detect veterinary drugs and pesticides in infant formula for safety and quality control purposes. This method incorporated 49 compounds belonging to 11 different compound classes.^67^

For the profiling and authentication of Ginseng in traditional Chinese medicine, Zhang *et al*. used ion mobility spectrometry (IMS) for the development of a UHPLC‐IMS‐Q‐TOF‐MS method for the added orthogonal separation of ginsenosides to more confidently distinguish the peaks of interest. For method optimization, the group investigated the effects of changing the tolerance (low/high) of both the CCS filtering for the ion mobility cell and *m/z* filtering for the quadrupole to effectively identify and avoid interfering substances (such as isomers) in the final method conditions. This dual filtering shows a potential for aiding the deconvolution of complex matrices and increasing method selectivity. The method conditions were validated and characteristic spectral profiles of the seven Ginseng drugs were generated through analyzing 60 batches of Ginseng. This method allows the simultaneous identification and differentiation of seven Ginseng drugs, which could have a direct impact on Chinese medicine quality standards and counterfeits.^68^

Wang *et al*. developed a comprehensive UHPLC‐Q‐Orbitrap‐MS method to separate and identify the related substances in polysorbates, which are commonly used pharmaceutical excipients. Through analyzing ten species in polysorbate‐80 such that 211 components were identified, the group used mathematical modeling to predict all components possible in polysorbates‐80, ‐60, ‐40, and ‐20 to create a database of 853 compounds for collective use.^69^ The database will prove useful for pharmaceutical product development and quality assessments of polysorbate materials.

Wu *et al*. developed a new UHPLC‐Q‐IT‐Orbitrap‐MS method for the identification and quantification of the structurally related impurities of calcitonin salmon (sCT), which is a pharmaceutical peptide drug approved in 2005 primarily used for the treatment of osteoporosis and hypercalcemia. With this method, the USP and EP reference standard impurities were accurately quantified, but several unreported major impurities were observed in the same reference standards above the > 1 mg/g reporting limit. Other minor impurities were also discovered due to the high sensitivity of HRMS. Some of these new impurities had been created during synthesis due to side‐chain reactions, replacement errors, and diastereoisomerization. This work shows the importance of using new technologies on legacy drug projects as the advances in HRMS allow a better understanding of drugs and their impurities that was not available before. Being able to detect the low‐level impurities helps ensure patient safety.^70^ Similarly, this group performed the same type of experiments for arginine vasopressin, which is a cyclic peptide drug to treat diabetes insipidus and esophageal varices bleeding.^71^

Brinkman *et al*. used GC‐TOF‐MS and UHPLC‐Q‐TOF‐MS to compare the excreted levels of asthma medication in exhaled breath and urine samples, respectively, in severe asthma patients. The study showed a link between systemic biomarkers of drug uptake in the urine and exhaled breath that could be replicated and validated. As both samples were shown to be useful to monitor recent drug intake and effect, this study demonstrated breathomics as a viable quick and non‐invasive point‐of‐care tool.^72^ This work shows how instrument advancements allow new means of testing, which could have a wider impact on how clinical trials are performed and could bring medicines to market faster.

### 6.3 Nitrosamines

Since 2018, there has been a lot of focus on nitrosamines, chiefly *N*‐nitroso‐dimethylamine (NDMA), *N*‐nitroso‐diethylamine (NDEA), and *N*‐nitroso‐*N*‐methyl‐4‐aminobutyric acid (NMBA),^73^ as impurities in medicines due to their acute carcinogenicity. The acceptable intake is currently set at less than 100 ng/day^73^ and due to this low limit, several drug products, including angiotensin receptor blockers, histamine blocker ranitidine, and metformin, have had to be recalled from the market, resulting in large losses for the companies involved.

**FIGURE 5:**
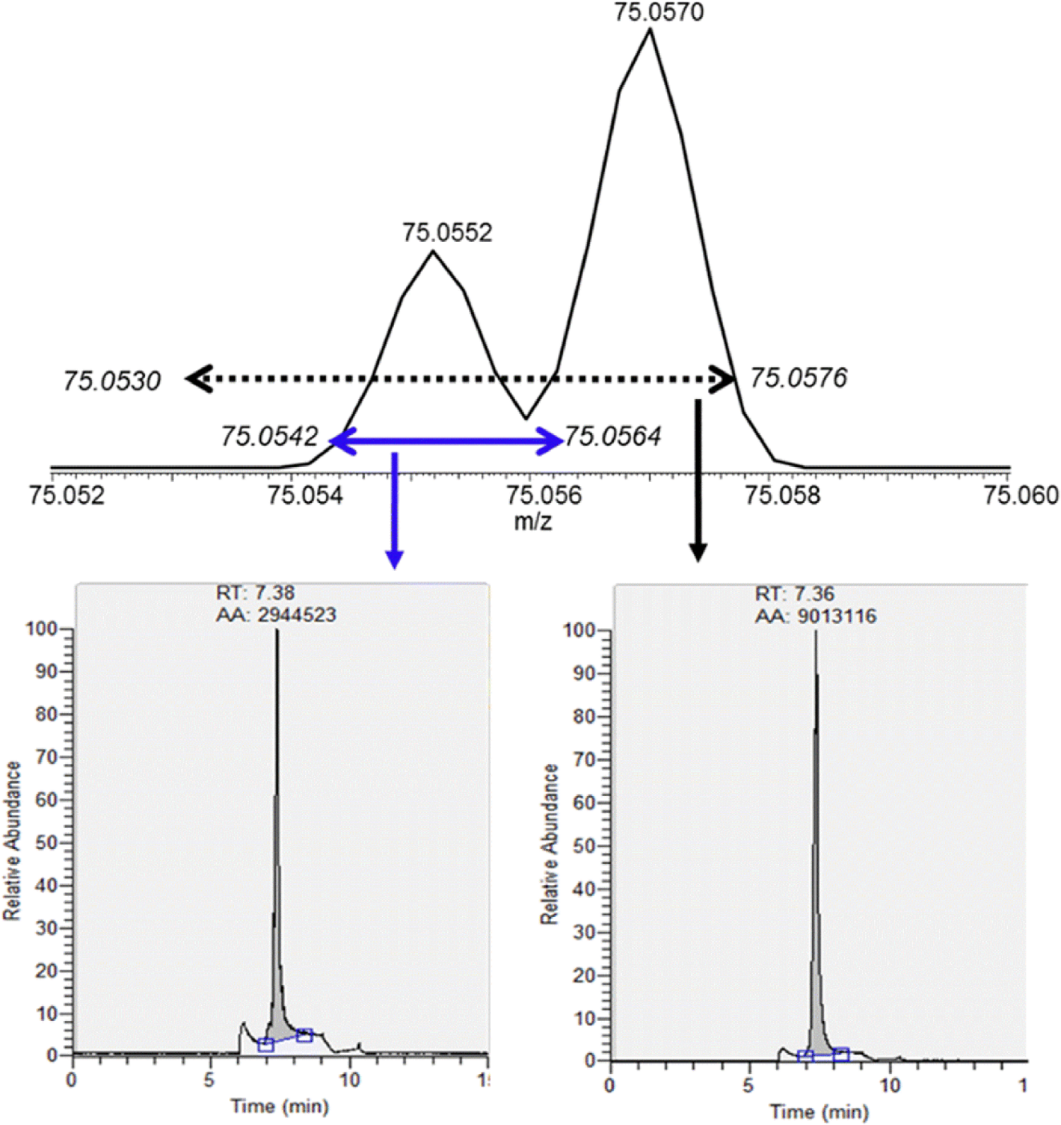
Mass spectrum of metformin drug product spiked with NDMA (20 ng/mL) that also contained DMF, where the protonated NDMA ion is observed at *m/z* 75.0552 and the DMF ^15^N‐isotopic peak is at *m/z* 75.0570. Reconstructed ion current chromatograms demonstrate the overestimation of NDMA (by including the DMF isotopic interference) through using a wide mass tolerance setting: (left; blue) ± 15 ppm, (right; black) ± 30 ppm. Reprinted with a permission from^75^

In collaboration with pharmaceutical companies, the FDA developed, validated, and made publicly available a UHPLC‐HRMS method for the detection and quantitation of NDMA in metformin drug products.^74^ Recently, high levels of NDMA above the acceptable limits were reported for 16 of 38 metformin products using this method by a private laboratory in a Citizen Petition file.^75^ To confirm these findings, the FDA tested the 38 lots using three orthogonal methods with one being the same method used as the private laboratory, however, only 8 were found to be above the limit. Generally, for all the same samples, the FDA analysis measured significantly lower levels of NDMA than the private laboratory. Yang *et al*. described the findings of the laboratory investigation, where it was found that the UHPLC‐Q‐TOF mass spectrometer used in the private laboratory had insufficient mass resolution or accuracy or inappropriate mass tolerance settings in data processing to separate the interfering DMF ^15^N‐isotope from NDMA, leading to an overestimation of NDMA (Figure 5). The added resolving power of the UHPLC‐Q‐Orbitrap mass spectrometer used by the FDA avoided this error. This work highlights the importance of adequate HRMS performance for its analytical purposes to avoid false positives, especially in this case where the product recalls would have incurred huge losses and reputational damage for the pharmaceutical companies and denied patients their medicines.^75^

### 6.4 Environmental monitoring

Unfortunately, with the increased production and consumption of pharmaceuticals, the presence of drugs in the environment has grown. These are included in the umbrella term “emerging pollutants” (EPs), which are synthetic or naturally occurring compounds released into the environment that are not monitored. These substances enter the aquatic environment mainly through wastewater treatment plant (WWTP) discharges due to either partial or nonexistent removal during wastewater treatment.^76^ There is a concern that these compounds could negatively impact ecology due to adverse effects and/or persistency^77^; therefore, there is a demand for analytical methodologies that can control and monitor a large number of compounds as a means to evaluate water quality. Environmental samples constitute complex matrices that make analytical method development and validation a challenging task. In 2020, many HRMS screening methodologies have been developed to detect pollutants in water that are ‘fit for purpose’, *i.e*. sensitive, selective, and comprehensive. For instance, Gago‐Ferrero *et al*. have developed and validated a UHPLC‐Q‐TOF‐MS method capable of screening 2316 EPs in water.^78^ Tian *et al*. used HRMS to detect and quantify 87 EPs in the marine environment of Puget Sound.^79^

Vermeulen *et al*. discuss the advances of the “exposome” concept, which was conceived in 2005 to represent the environmental factors and influences on the human phenotype. This area of research is not well understood despite having large implications on human health across the world (and by extension the economic costs *via* healthcare). Nine million deaths per year (16% of all death worldwide) were attributed to air, water, and soil pollution alone.^80^ This number is likely to be grossly underestimated due to the lack of understanding and testing of EPs in the environment.^81^ Due to recent technological advancements, it is now possible to perform the systematic mapping of both endogenous and exogenous species in the environment required to study the exposome, especially with HRMS and its advantages for screening methods. The paper further explores how the use of unbiased HRMS screening and potential high‐throughput identification of compounds through literature mining offers the ability to perform large‐scale exposomic data analyses and further understand the impact of our environments on our bodies.^81^ This insight will prove invaluable to define the future environmental and social responsibilities required from pharmaceutical companies regarding their products.

## 7 CONCLUSION

The advances in MS technology, in particular HRMS, combined with the wider accessibility of instrumentation across the scientific community, has manifested itself in the greater adoption of the technique across pharmaceutical drug development for both qualitative and quantitative purposes. The strengths of HRMS make it a game‐changer for routine MS characterization and global screening applications, where the performance characteristics of HRMS allow (i) more confident compound identifications through accurate mass measurements and isotope patterns, (ii) better overall ion separation and detection, (iii) quantification on par with QqQ using SRM acquisition, and (iv) the ability to create rapid, comprehensive screening methodologies that can be applied to complex samples. HRMS instrumentation directly challenges the traditional LRMS setups and it will be likely to replace these for many laboratories in the coming years.

In recent years, the approaches taken within pharmaceutical development have evolved to include novel drug modalities and whole biological system analyses, which often require large HRMS data sets and 'big data'. Manual processing of these data sets would be time‐consuming and laborious, which means automated data processing *via* software becomes necessary to enable high‐throughput workflows. Several groups have focused on evaluating currently available software and compiling databases for various applications, and this reflects the current status in this area, namely that these software and their associated workflows are constantly being conceived, developed, and improved. This is an exciting area of development, where laboratories worldwide are leading collaborative efforts for collective use, and new innovative approaches, such as the use of artificial intelligence and machine learning, are being explored. These initiatives offer great potential to maximize the knowledge gained from HRMS data, which will contribute significantly to the discovery and development of more efficacious medicines in shorter timeframes, which benefits patients worldwide.
